# Genome Analysis of a Transmissible Lineage of *Pseudomonas aeruginosa* Reveals Pathoadaptive Mutations and Distinct Evolutionary Paths of Hypermutators

**DOI:** 10.1371/journal.pgen.1003741

**Published:** 2013-09-05

**Authors:** Rasmus Lykke Marvig, Helle Krogh Johansen, Søren Molin, Lars Jelsbak

**Affiliations:** 1 Department of Systems Biology, Technical University of Denmark, Lyngby, Denmark; 2 Department of Clinical Microbiology, Rigshospitalet, Copenhagen, Denmark; Uppsala University, Sweden

## Abstract

Genome sequencing of bacterial pathogens has advanced our understanding of their evolution, epidemiology, and response to antibiotic therapy. However, we still have only a limited knowledge of the molecular changes in *in vivo* evolving bacterial populations in relation to long-term, chronic infections. For example, it remains unclear what genes are mutated to facilitate the establishment of long-term existence in the human host environment, and in which way acquisition of a hypermutator phenotype with enhanced rates of spontaneous mutations influences the evolutionary trajectory of the pathogen. Here we perform a retrospective study of the DK2 clone type of *P. aeruginosa* isolated from Danish patients suffering from cystic fibrosis (CF), and analyze the genomes of 55 bacterial isolates collected from 21 infected individuals over 38 years. Our phylogenetic analysis of 8,530 mutations in the DK2 genomes shows that the ancestral DK2 clone type spread among CF patients through several independent transmission events. Subsequent to transmission, sub-lineages evolved independently for years in separate hosts, creating a unique possibility to study parallel evolution and identification of genes targeted by mutations to optimize pathogen fitness (pathoadaptive mutations). These genes were related to antibiotic resistance, the cell envelope, or regulatory functions, and we find that the prevalence of pathoadaptive mutations correlates with evolutionary success of co-evolving sub-lineages.

The long-term co-existence of both normal and hypermutator populations enabled comparative investigations of the mutation dynamics in homopolymeric sequences in which hypermutators are particularly prone to mutations. We find a positive exponential correlation between the length of the homopolymer and its likelihood to acquire mutations and identify two homopolymer-containing genes preferentially mutated in hypermutators. This homopolymer facilitated differential mutagenesis provides a novel genome-wide perspective on the different evolutionary trajectories of hypermutators, which may help explain their emergence in CF infections.

## Author Summary

*Pseudomonas aeruginosa* is the dominating pathogen of chronic airway infections in patients with cystic fibrosis (CF). Although bacterial long-term persistence in CF hosts involves mutation and selection of genetic variants with increased fitness in the CF lung environment, our understanding of the within-host evolutionary processes is limited. Here, we performed a retrospective study of the *P. aeruginosa* DK2 clone type, which is a transmissible clone isolated from chronically infected Danish CF patients over a period of 38 years. Whole-genome analysis of DK2 isolates enabled a fine-grained reconstruction of the recent evolutionary history of the DK2 lineage and an identification of bacterial genes targeted by mutations to optimize pathogen fitness. The identification of such pathoadaptive genes gives new insight into how the pathogen evolves under the selective pressures of the host immune system and drug therapies. Furthermore, isolates with increased rates of mutation (hypermutator phenotype) emerged in the DK lineage. While this phenotype may accelerate evolution, we also show that hypermutators display differential mutagenesis of certain genes which enable them to follow alternative evolutionary pathways. Overall, our study identifies genes important for bacterial persistence and provides insight into the different mutational mechanisms that govern the adaptive genetic changes.

## Introduction

A molecular and mechanistic understanding of how bacterial pathogens evolve during infection of their human hosts is important for our ability to fight infections. The advent of high-throughput sequencing techniques now offer unprecedented nucleotide resolution to determine the relatedness among infecting bacterial isolates and to unveil genetic adaptation within infected individuals and in response to antibiotic therapy [1]–[7]. Unraveling the genetic content of pathogens helps to identify the genes that make certain bacterial lineages more pathogenic than others. Nonetheless, the pathogenicity of a bacterial clone can also evolve via the mutational changes of pre-existing genes, a mechanism which is also known as pathogenicity- or pathoadaptive mutations [8]. While several studies have provided insight into the genomic evolution of primary bacterial pathogens such as *Yersinia pestis* [6] and *Vibrio cholera* [2] causing acute infections, only little is known how these observations relate to opportunistic pathogens causing long-term infections [1].

The opportunistic pathogen *Pseudomonas aeruginosa* is a common environmental inhabitant, which is also capable of causing both acute and chronic infections in a range of hosts from amoeba and plants to humans. For example, *P. aeruginosa* causes chronic airway infections in most patients with cystic fibrosis (CF), and is directly associated with the morbidity and mortality connected with this disease. Chronic CF infections provide an opportunity for long-term monitoring of the battle between the infecting bacteria and the host immune defense and clinical intervention therapy [9], [10], and thus offer a direct method for observing evolutionary mechanisms *in vivo*. In an effort to understand the evolutionary mechanisms facilitating the transition of *P. aeruginosa* from its environment to a human host, we have previously found no evidence for horizontal acquisition of genes to play a role [9]. Instead, we suggested the establishment of long-term chronic infections to be a matter of tuning the existing genome via pathoadaptive mutations.

The within-host mutation rate is a key factor in determining the potential for bacterial pathogens to genetically adapt to the host immune system and drug therapies, and knowledge about *in vivo* growth dynamics of bacterial pathogens and their capacity for accumulation of mutations is essential for the design of optimal interventions. Interestingly, the generation of mutations is frequently accelerated in clinical populations of *P. aeruginosa* that evolves as so-called ‘hypermutators’ due to deficient DNA mismatch repair systems [11]. Although the hypermutable phenotype is also observed for other species in a range of conditions [12]–[16], the impact of this phenotype in a natural environment and in relation to infections remains less clear.

Here we analyze the genome sequences of 55 isolates of the transmissible *P. aeruginosa* DK2 clone type causing chronic infections in a cohort of Danish CF patients. Our collection, comprising both normal (normomutator) and hypermutable isolates, enabled a comparative analysis of evolutionary trajectories of individual sub-lineages of the DK2 clone type making it possible to identify genes targeted by pathoadaptive mutations. Furthermore, the long-term population dynamics and structure of the clonal expanding DK2 lineage was elucidated by a high-resolution phylogeny, and an examination of the mutation dynamics of homopolymers (homopolymeric tracts of identical nucleotides, *e.g.* GGGGG) provided novel genome-wide evidence for the potential advantage of differential mutagenesis associated with the hypermutator phenotype.

## Results and Discussion

### Strain collection and maximum-parsimonious phylogeny

**Figure 1:**
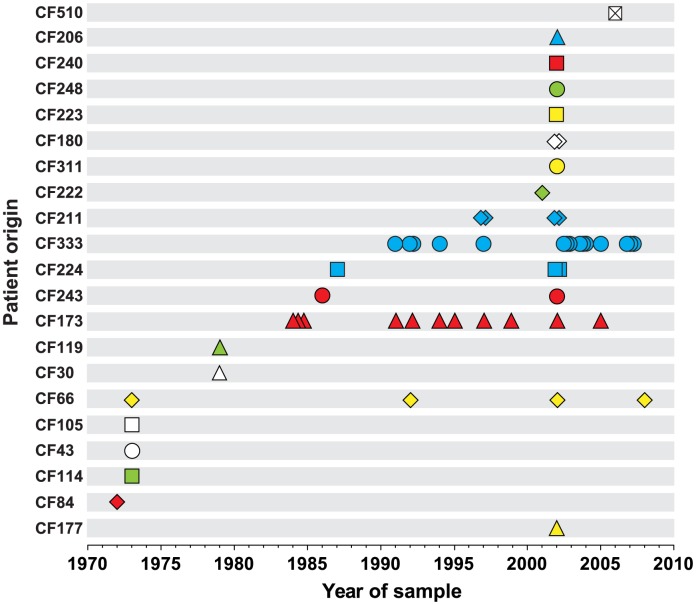
Patient origin and sampling time of genome sequenced *P. aeruginosa* DK2 isolates. The collection of 55 *P. aeruginosa* isolates of the DK2 clone type was sampled from 21 different CF patients over 38 years. Bacterial isolates are indicated by symbols, and if multiple isolates were sampled the same year from a patient, they are represented by stacked symbols. The isolates are named from the patient from whom they were isolated, and their isolation year (*e.g.* isolate CF173-1991).

We sequenced the genomes of a collection of 55 *P. aeruginosa* DK2 clones sampled from Danish CF patients between 1972 and 2008 (Figure 1 and Table S4). The sequence data of 45 of the isolates have previously been reported [9], [10]. Most patients (n = 19) were represented by only a single or a few (≤4) isolates. However, two patients were represented by 11 and 15 isolates (CF173 and CF333, respectively).

**Figure 2:**
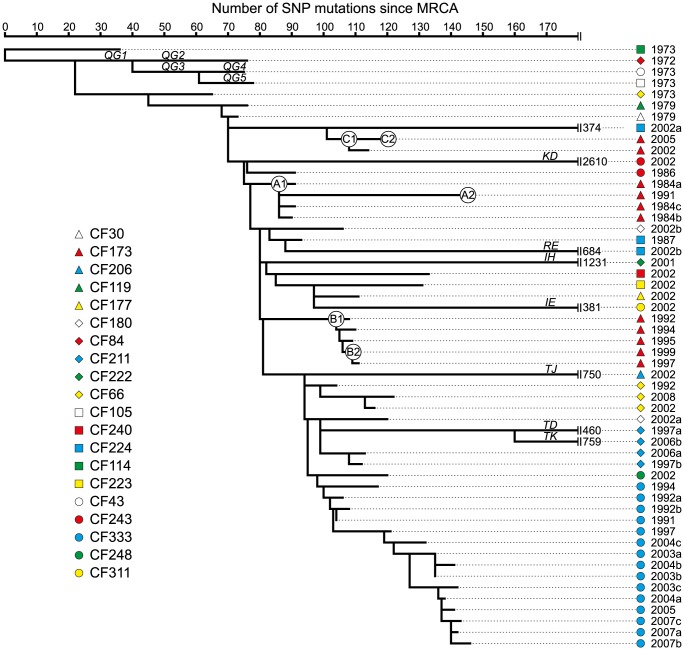
Maximum-parsimonious reconstruction of the phylogeny of the *P. aeruginosa* DK2 clones. The phylogenetic tree is based on 7,326 SNPs identified from whole-genome sequencing, and lengths of branches are proportional to the number of mutations. Outlier isolate CF510-2006 [9] (not shown) was used as an outgroup to determine the root of the tree. Branches leading into *mutS*, *mutL*, or, *mutY* hypermutable isolates are named as indicated by italic letters. Statistics on mutations accumulated in the specific branches are summarized in Table 1. Circles labeled A1, A2, B1, B2, C1, and C2, respectively, denotes the position of the first and last genotype of each of the DK2 sub-lineages A, B, and C which were observed to have infected patient CF173.

We identified a total of 7,326 unique SNPs in the 55 DK2 genomes, that could be explained by 7,368 mutational events (consistency index 0.99) using a maximum-parsimonious phylogenetic model to elucidate the evolutionary relationship of the *P. aeruginosa* DK2 population (Figure 2). The high consistency of the tree reflects the unidirectional, clonal evolution from the root of the tree to the tips, thus enabling inferences about the succession of mutations and the relationship among *P. aeruginosa* DK2 clones.

### Prevalence of mutS, mutL, and mutY hypermutators

**Figure 3:**
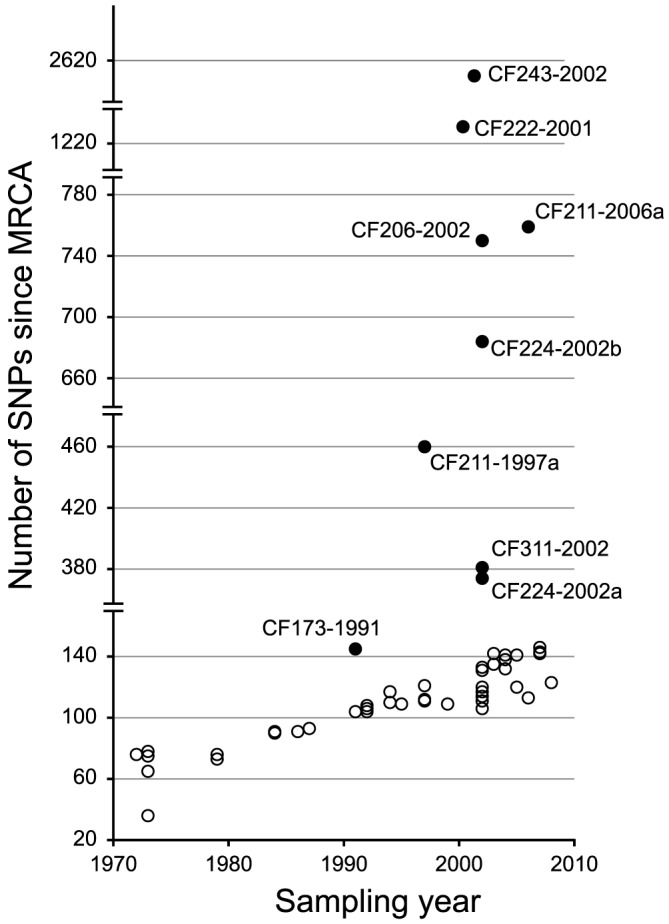
Total number of SNPs accumulated in each DK2 isolate. The number of SNPs accumulated in each of the isolates since their most recent common ancestor (MRCA) is plotted against isolate sampling year.

From the phylogenetic tree we observed a linear correlation between the number of SNPs and the time of sampling (*i.e.* a constant rate of mutation accumulation during the clonal expansion of the DK2 lineage) (Figure 3). However, nine sub-lineages (indicated by filled circles in Figure 3) deviated from this trend and had accumulated mutations at higher rates. In one of these isolates, CF224-2002a, we found that 265 of the 273 SNPs accumulated in the branch leading to the isolate were densely clustered in two chromosomal regions with SNP densities (1.2 and 1.8 SNPs per kb, respectively), that are much higher than expected (0.043 SNPs per kb assuming a random distribution of SNPs) (Figure S1). The most likely explanation for these high SNP densities is that the two genomic regions are the result of recombination events with DNA from a *P. aeruginosa* strain(s) unrelated to the DK2 clone type. Another study by Chung *et al.* observed similar indications of within-patient recombination events in *P. aeruginosa* [17].We found no evidence for additional recombination events among the 55 genome sequences.

The excess numbers of mutations in the remaining eight deviant isolates were the result of increased mutation rates due to mutations in mismatch repair and error prevention genes. Seven isolates had non-synonymous mutations in one of the DNA mismatch repair (MMR) genes *mutS* (n = 2) and *mutL* (n = 4) or both (n = 1), and their excess numbers of SNPs showed a highly increased transition∶transversion ratio consistent with MutS or MutL defects (Table 1) [3], [11], [17]–[19]. Moreover, one isolate (CF173-1991) had a mutation in *mutY* and a molecular signature consistent with a MutY defect (*i.e.* a high proportion of transversions) (Table 1).

**Table 1:** Statistics on mutations accumulated in hypermutable sub-lineages.

Branch^a^	Clone at tip of branch	*mutS*, *mutL*, and, *mutY* mutations^b^		SNPs	Transitions∶Transversion ratio	Microindels (% within homopolymers)
TJ	CF206-2002	*mutL(1248–1249insGCGCC)*		656	54∶1	142 (90%)
TD	CF211-1997a	*mutL(C1420T)* c	MutL(R474W)	300	59∶1	79 (94%)
TK	CF211-2006a	*mutL(C1420T)* c	MutL(R474W)	599	53∶1	114 (82%)
IH	CF222-2001	*mutS(T347C; 1096–1097insC; G1561A)*	MutS(V116A; A521T)	1149	43∶1	219 (90%)
RE	CF224-2002b	*mutL(792–802ΔTATGGTGCGCG; C1477T)*	MutS(P493S)	596	65∶1	126 (86%)
KD	CF243-2002	*mutS(G506A; G1300A; C1495A), mutL(T875C; G1452A)*	MutS(R169H; E434K; R499S), MutL(V292A; M484I)	2534	86∶1	268 (75%)
IE	CF311-2002	*mutS(G1567A)*	MutS(E523K)	284	40∶1	104 (94%)
QG1-5	CF84-1972, CF43-1973, CF105-1973	*mutY(G925A)*	MutY(V309M)	106	1∶5	11 (9%)
JC	CF173-1991	*mutY(T785A)*	MutY(L262H)	59	1∶6	5 (20%)
	Other	None		1085	2∶1	312 (21%)

a Branch names are denoted in phylogenetic tree in Figure 2.

b Position of mutations (consequences at the amino acid level is only shown for SNP mutations).

c Mutation accumulated in an ancestor shared by clones CF211-1997a and CF211-2006a.

We did not find other mutations in *mutS* or *mutL* among the remaining genome sequences, but three additional early isolates (CF84-1972, CF43-1973, and CF105-1973) had mutations in *mutY* as well as having the molecular signature associated with a MutY defect (Table 1).

In total, we found 11 hypermutator strains among the 55 isolates. These mutators were found in ten of the 21 patients in our study (48%), which is comparable to previous findings (36%) [11]. Our results include two patients (CF211 and CF224) from whom we isolated both hypermutators and normal (normomutator) clones documenting the co-existence of both types. Indeed, the identification of both a hypermutable and a normal sub-lineage in years 1997 and 2006 from patient CF211 suggests at least 9 years of co-existence within this patient (Figure 2). It is possible that the sub-lineages with different mutation rates occupy different niches within the hosts, each niche representing different selection pressures.

### Mutation rate of homopolymers

We next designed our mutational analysis to detect small insertions and deletions (microindels). A total of 1,204 unique microindels were discovered. The inheritance was explained by 1,380 parsimonious events and was congruent with the SNP-based phylogeny although the consistency for the microindels was lower (0.87) than for the SNPs (0.99). The higher rate of homoplasy among microindels would a priori be expected as microindels accumulate with high rates at mutational hotspots consisting of simple sequence repeats (SSRs) [20]. Accordingly, 93% of the inconsistent microindels were located in SSRs. As expected from current knowledge [21], [22], we observed that the seven *mutL/mutS* hypermutators were particularly prone to mutation within SSRs consisting of homopolymers, and as a result 86% of the microindels that accumulated in the *mutS*/*mutL* hypermutable sub-lineages were localized in homopolymers whereas this was only true for 21% of the microindels within the remaining sub-lineages (Table 1).

Highly mutable loci have been shown to be important for pathogenesis and host adaptation of several pathogens [23]. For example, increased mutation rates of homopolymers in MMR-deficient *P. aeruginosa* strains have been shown *in vitro* to be important for mutational inactivation of the regulatory gene *mucA* [24], which is pivotal for adaptation in CF airways. Nonetheless, we only have a limited understanding of the homopolymer mutation dynamics at a genome-wide level and of the impact of increased mutation rates of homopolymers in relation to host adaptation. However, our collection of genome sequences from both normal and hypermutator isolates, sampled from the airways of CF patients, provides an opportunity to shed new light on homopolymer mutation rates and their impact on adaptation.

**Figure 4:**
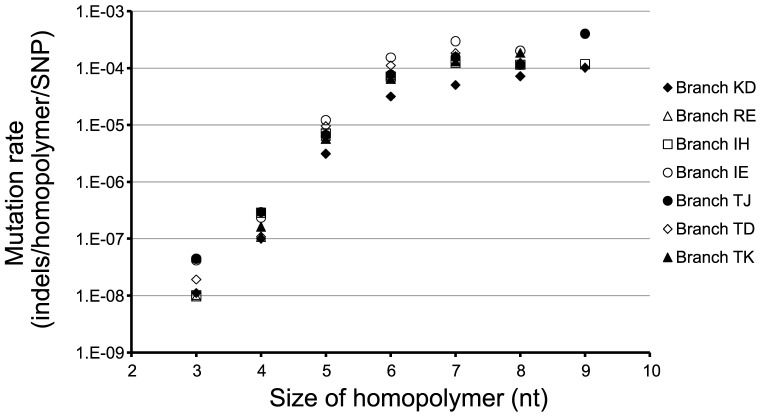
Mutation rates of homopolymers. Rates of mutation of homopolymers of different sizes are shown for seven DK2 sub-lineages evolving with a *mutS/mutL* DNA MMR-deficiency. The rates were calculated as the number of observed indels per homopolymer per *mutS/mutL* MMR-deficiency caused SNP (see Materials and Methods).

For each of the seven *mutS/mutL* hypermutator sub-lineages we calculated the mutation rates of homopolymers of different lengths (Figure 4). We observed that longer homopolymers were more likely mutated than homopolymers of shorter lengths, and for homopolymers of 3–6 nucleotides length the mutation rate increased exponentially (*R* = 0.995; Student's *t*-test, *P* = 0.0026). One might expect large homopolymers to exhibit higher probabilities of mutation, because they are distributed more frequently outside coding regions. However, we observed no evidence of this playing a role, as mutation rates of intergenic and intragenic homopolymers were similar (Figure S2). Instead, the size-dependent mutation rate of homopolymers is likely to be a consequence of the mechanistically determined probabilities of strand-slippage during replication [20].

### Distinct evolutionary paths in hypermutators

The size-dependent mutation rates of homopolymers of different lengths suggest that different genes have different probabilities of mutation. In this way, certain genes, in which variation is appreciated, may harbor sequences that are more frequently mutated in contrast to essential genes in which genetic changes are strongly selected against [23]. In agreement with this, we find that genes annotated as essential genes in *P. aeruginosa* PAO1 [25] are less likely to contain large homopolymers ≥7 nt (Fisher's exact test, *P* = 0.037) (Table S1).

Survival of bacteria in human hosts has previously been suggested to be positively influenced by rapid modulation of the cell envelope. In agreement with this (and in opposition to essential genes), we find that genes functionally related with the composition of the cell envelope are more likely to contain large ≥7 nt homopolymers (Fisher's exact test, *P* = 0.002) (Table S1). This leads us to speculate that hypermutators have a selective advantage over their normal counterparts, not only because they can speed up evolution, but also because they are creating a bias towards a different evolutionary path by homopolymer facilitated differential mutagenesis.

In support of our hypothesis, we find that *mutS/mutL* hypermutators acquire 3.7 more mutations in cell envelope genes containing large homopolymers (≥7 nt) relative to cell envelope genes without large homopolymers, and that the accumulation of mutations in the homopolymer-containing cell envelope genes is due to mutations within the homopolymers. Accordingly, 50% of mutations in homopolymer-containing cell envelope genes are indels whereas this is only true for 5% of the mutations in the remaining genes (9/164 vs. 8/8; Fisher's exact test, *P* = 6.2×10^−6^).

In further support of our hypothesis on differential mutagenesis we find two genes (PADK2_15360 and PADK2_03970) in which all seven *mutS*/*mutL* hypermutators, but no other isolates, carries mutations. Given the number of mutations within each of the seven hypermutable sub-lineages and all other lineages this observation is highly unexpected by chance (*P(X*≥*2)∼binom(X; 5976; 2.9*×*10^−7^)* = 1.6×10^−6^; where 2.9×10^−7^ is the probability of an average length gene to be mutated in only the *mutS*/*mutL* sub-lineages). One of the genes, PADK2_15360, encodes an outer membrane receptor protein, and all seven hypermutators are independently mutated in the same 7×G homopolymer located at position 1127–1133 within the 2958 nt gene. Since none of the other 48 isolates contain mutations within PADK2_15360, we suggest that mutations in this gene represent a hypermutator-specific adaptive target for rapid modulation of the cell envelope. All seven mutations are frameshift mutations causing premature stop codons resulting in truncated proteins without a putative TonB dependent receptor domain (Pfam family PF00593) located in the C-terminal part of the protein. We hypothesize that this domain is localized in the outer membrane where it, due to its potential surface-exposure, could be a target of recognition by the immune defense.

### Time-measured phylogenetic reconstruction of the DK2 lineage

**Figure 5:**
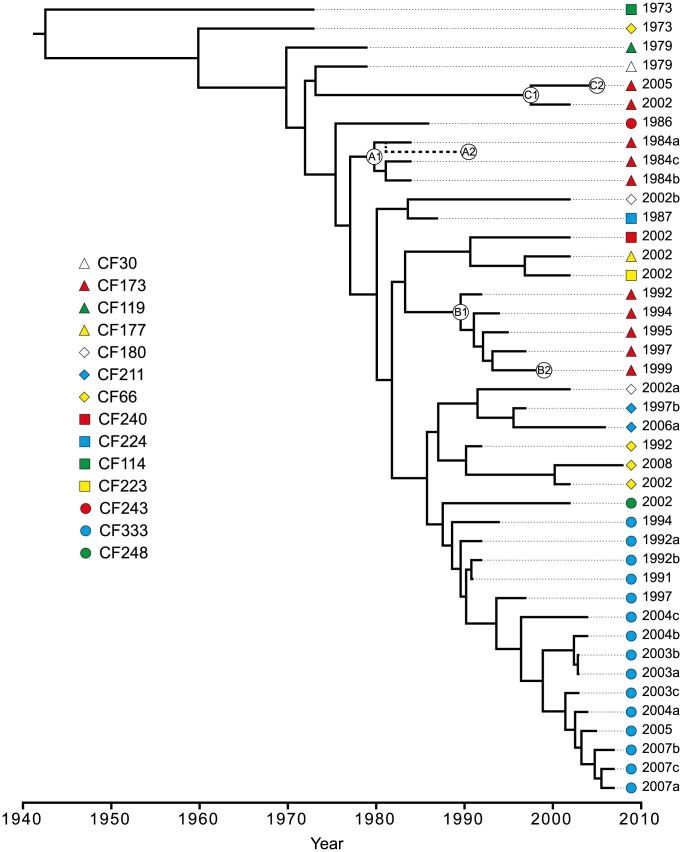
Bayesian phylogenetic reconstruction and divergence date estimates of the *P. aeruginosa* DK2 clones. Bayesian statistics were used to estimate the divergence times of predicted ancestors. The tree was based on 736 unique SNPs identified from whole-genome sequencing. Circles labeled A1, A2, B1, B2, C1, and C2, respectively, denotes the position of the first and last genotype of each of the DK2 sub-lineages A, B, and C which were observed to have infected patient CF173. The position of CF173-1991 (A2) is approximated from an equivalent Bayesian phylogenetic analysis including the hypermutator isolates (Figure S3).

To further investigate the within-host evolutionary history of the DK2 lineage and to estimate the dates of divergence between DK2 isolates, we applied Bayesian statistics to infer time-measured phylogenies using a relaxed molecular clock rate model (Figure 5).

We excluded the hypermutator isolates and isolate CF224-2002a containing recombined regions from the analysis, as they would otherwise interfere with the phylogenetic analysis. Based on this analysis, the mean mutation rate was estimated to be 2.6 SNPs/year (95% highest posterior density (HPD; see Materials and Methods) 1.8–3.2 SNPs/year) which is equivalent to 4×10^−7^ SNPs/year per site or 9×10^−11^–11×10^−11^ SNPs/bp per generation assuming 3700–4500 generations per year [26]. Our estimated mutation rate is in the same range as those estimated for *Shigella sonnei* (6×10^−7^ SNPs/bp/year) [7] and *Vibrio cholerae* (8×10^−7^) [2] but in between the rates reported for *Yersinia pestis* (2×10^−8^) [6] and *Staphylococcus aureus* (3×10^−6^) [27].

The topologies of the Bayesian phylogenetic reconstruction and the maximum-parsimonious phylogeny were congruent, and the relationship among the clones correlated with patient origin and the time of sampling (Figure 2; Figure 5).

We have previously shown that a set of specific mutations first observed in CF30-1979 and in all isolates sampled after 1979 were important for the reproductive success of the DK2 lineage and its dissemination among multiple individuals [10]. Using the phylogenetic reconstruction, we estimate that isolates sampled after 1979 diverged from a common ancestor in year 1970 (95% HPD, 1961–1976) [10]. Furthermore, our phylogenetic data document that the transmission potential of the DK2 lineage has been maintained over several decades. The most recent transmission event is predicted to have occurred in year 1997 (95% HPD, 1991–2001), as this is the latest time estimate of a predicted ancestor shared by isolates from different patients (CF177-2002 and CF223-2002). Since we have not investigated DK2 isolates from all patients chronically infected with this lineage it remains a possibility that transmission has occurred subsequent to this time.

Seven patients are represented by multiple isolates, and in six of the patients at least two of the isolates clustered as monophyletic groups according to patient origin (Figure 2; Figure 5). This is in agreement with a model in which independent sub-lineages of the DK2 clone evolved separately within individual patients, and it excludes the possibility of continuous and near-perfect mixing of strains between patients. The patient-linkage was most prevalent for patient CF333 from which all 15 isolates constituted a single monophyletic group, and the isolates branched in general according to their sampling year giving a linear evolutionary trajectory with an average distance of 6.1 SNPs (∼2.3 years) from the line of descent (Figure 2; Figure 5).

In contrast, we observed an unexpected DK2 population dynamics in patient CF173 in which the isolates clustered as three different monophyletic groups with four, five and two isolates, respectively (Figure 2). This shows that patient CF173 was infected by three distinct sub-lineages rather than only a single sub-lineage. Interestingly, the three sub-lineages carried by patient CF173 can be distinguished based on the sampling year of the isolates. Accordingly, the isolates from the different clusters are sampled in the time-periods 1984–1991 (cluster A), 1992–1999 (cluster B) and 2002–2005 (cluster C), respectively. This points to a replacement of the earlier sub-lineages around years 1991–1992 and 1999–2002, respectively, caused by secondary transmission events. Alternatively, it could be the result of co-existing lineages whose time-dependent sampling was caused by shifts in relative abundance or changes in sampling probability from different niches.

### Parallel evolution of genes involved in host adaptation

**Figure 6:**
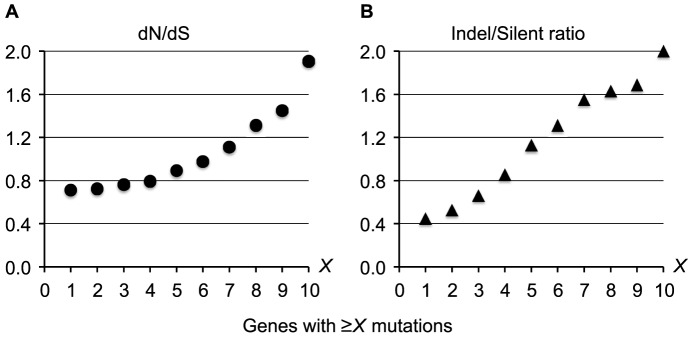
Increased pressures of selection for mutations in the top most mutated genes. Measures of the selection pressures were plotted for genes acquiring ≥*X* mutations during the evolution of the DK2 lineage. Plot A shows the dN/dS ratio, and plot B shows the ratio of indels relative to silent SNPs.

The presence of independently evolving DK2 sub-lineages made it possible to search for recurrent patterns of mutation and to identify bacterial genes that have acquired mutations in parallel in different individuals [1], [28]. Overall, we found no evidence for either intragenic bias of the mutations or for positive selection within coding regions (dN/dS = 0.66 including all mutations; Text S1), and we would therefore expect the 7,383 intragenic mutations to be distributed randomly among the 5,976 *P. aeruginosa* DK2 genes. This means that on average a gene would acquire 1.2 mutations, and we would expect only 1.3 genes to acquire mutations more than 6 times (*P(X>6)∼binom(X; 7,383; 5976^−1^)* = 2.2×10^−4^). Nonetheless, we identified 65 genes that were mutated more than 6 times when comparing across all DK2 sub-lineages (see Table S2 for the full list of all 65 genes). The high mutation number within these genes could be the result of a positive selection for mutations, which is supported by our observation that increased pressures of selection acts on the top most mutated genes (Figure 6). Accordingly, the signature for selection for SNPs accumulated in the 65 top most mutated genes (dN/dS = 1.11) was positive and significantly higher than for SNPs accumulated in other genes (dN/dS = 0.69; Fisher's exact test, *P* = 5.2×10^−5^).

These findings suggest that the 65 genes with multiple mutations undergo adaptive evolution (*i.e.* they are pathoadaptive genes involved in host adaptation), although the presence of neutral mutational hotspots or fast acquisition of secondary mutations within the same gene may contribute to the high mutation number in some genes. To exclude the possibility that the high mutation numbers were the result of recombination events or because of particularly large gene sizes, we left out mutations from recombined regions and large genes (>5 kb) from our analysis.

**Figure 7:**
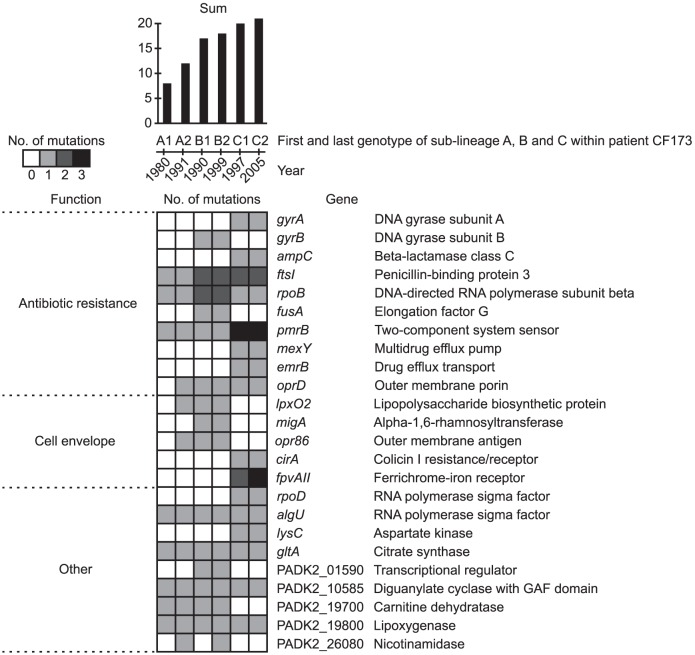
Pathoadaptive genes. Genes identified from parallel evolution to be involved in host adaptation. Colors of squares denotes if the gene was mutated relative to the MRCA of the DK2 clones. Only genes mutated in any of the isolates from CF173 are shown (see full list of pathoadaptive genes in Table S2). The presence of mutations is shown for the first and last genotypes of each of the DK2 sub-lineages A, B, and C, which were observed to have infected patient CF173. The total sum of mutations observed within each of the genotypes is indicated at the top. Genes are grouped by function. Details about specific mutations and their fixation in the DK2 isolates are given in Table S5, Table S6, and Figure S4.

A large part of the identified pathoadaptive genes were associated with antibiotic resistance (n = 14), including the genes *ampC*, *emrB*, *ftsI*, *fusA*, *gyrA/B*, *mexB/Y*, *pmrB*, *pprA*, *oprD*, and *rpoB/C* (Figure 7 and Table S2), in which mutations have been shown to confer resistance against a range of antibiotics, *e.g.* beta-lactams, tetracyclines, quinolones, chloramphenicol, macrolides, fusidic acid, aminoglycosides, polymycins and penicillins [29]–[37]. As such, the detection of multiple mutations in known antibiotic resistance genes confirmed the ability of our approach to identify genes involved in host adaptation. The exact amino acid changes caused by nine out of 16 unique non-synonymous mutations found within the genes *gyrA/B* and *rpoB* have previously been shown to confer resistance against fluoroquinolones and rifampicin, respectively (Table S3).

Another major group of pathoadaptive genes (n = 18) were functionally related to the cell envelope (Figure 7 and Table S2). Possibly, these mutations have been selected to evade the host immune response [38] or, especially in the case of *lpxO2*, to prevent interaction from LPS-targeting antibiotics [39]. Also, mutations in 13 genes involved in gene regulation were identified in our analysis, suggesting that remodeling of regulatory networks is a key evolutionary pathway in host adaptation as it seems to be in evolving *Escherichia coli* populations [40]. Among the regulatory genes that acquired mutations were four yet uncharacterized genes encoding components of two-component regulatory systems, a gene-category which is significantly overrepresented (88/5823 vs. 7/58 Fisher's exact test, *P* = 6.7×10^−5^) among the pathoadaptive genes (Table S2). We suggest that these uncharacterized regulatory genes as well as other genes identified as involved host adaptation represent potential therapeutic targets.

### Mutations in pathoadaptive genes correlate with strain displacement events within patient CF173

The adaptive benefits of a mutation are usually investigated by introduction of single or multiple mutations into isogenic strains and testing for fitness effects associated with the mutation(s) in controlled experimental conditions (such as competition experiments). Such testing is most effective when the phenotype (*e.g.* antibiotic resistance) can be easily interpreted in relation to the fitness impact. However, for mutations for which no or only subtle phenotypic changes are apparent it is difficult to directly test the fitness effects. In addition, the impacts on fitness of specific mutations must be assessed in the same environment as the one in which the mutation was selected. This is obviously not possible in case of human airway infections. To circumvent these limitations, we hypothesize that the count of mutations within the pathoadaptive genes can be used as a measure of the fitness of individual clones of *P. aeruginosa*. To investigate this hypothesis we took advantage of the two strain displacements (or changes in strain abundances) that occurred in patient CF173 in the years 1991–1992 and 1999–2002, which suggested that CF173 was infected by three succeeding DK2 sub-lineages A (1980–1991), B (1990–1999), and C (2000–2005).

We assume that the succeeding sub-lineage must be better adapted (*i.e.* having a higher fitness) than the previous sub-lineage, which was outcompeted. When determining the number of mutations found in the sub-lineages A, B, and C within the pathoadaptive genes, it was striking that the succeeding genotype consistently had a higher count of mutations than the previous genotype (Figure 7). In this way, the counts of mutations correlated with the strain displacement observed within patient CF173. We suggest that the mutation count can be used to predict the fitness of emerging DK2 clones, and that the pathogenicity scoring together with the information about the specific mutations can be used as a novel approach for clinicians to treat and segregate patients. It should be noted that our results cannot simply be ascribed to the succeeding genotypes having more mutations in general as no significant positive correlation existed between the total number of mutations and the number of mutations within the pathoadaptive genes (*R* = 0.30; Student's *t*-test, *P* = 0.28).

### Conclusions and implications

By genome sequencing of 55 isolates of the transmissible DK2 clone type of *P. aeruginosa*, we have provided a detailed view of the evolution of a bacterial pathogen within its human host. The sampling from multiple patients offered the opportunity to detect loci that were independently mutated in parallel lineages, here referred to as pathoadaptive genes, whereas sampling multiple times from the same patient gave an opportunity to study the within-patient population dynamics.

Several of the pathoadaptive genes identified here were associated with antibiotic resistance, gene regulation, and composition of the cell envelope. Some of these genes have been found in other studies of genomic evolution in CF pathogens to be important for adaptation [1], [3], [17]. Genomic analysis of additional *P. aeruginosa* lineages from different patients and clinical settings will enable a systematic identification of genes that are repeated targets for selective mutations during adaptation to life in the CF lung. Importantly, we also identified genes of unknown function and without prior implication in pathogenesis. Further investigations of the function of these genes are required to determine their potential as future therapeutic targets against the infection.

An exceptional 21-year time series of 11 isolates sampled from patient CF173 revealed a complex population dynamics in which the patient was infected by three distinct sub-lineages of the DK2 clone type, each sub-lineage being dominant over several years until its final decline or disappearance. This observation illustrates the power of high-throughput sequencing in relation to uncovering pathogen dynamics within infected individuals. We further observed that the cumulative count of mutations within pathoadaptive genes increased for each of the succeeding sub-lineages. This means that emerging sub-lineages carried a cumulative palette of pathoadaptive mutations and not only adaptive mutations conferring an advantage for a newly introduced selection force that may have triggered the removal of the preceding lineage.

The identification of pathoadaptive genes involved in host adaptation and our finding that the specific count of mutations within these genes act as a classifier that predict the pathogenicity of emerging sub-lineages of the DK2 clone type, should enable better epidemiological predictions and provide valuable information for the clinicians on how to treat and segregate patients.

The presence of hypermutable lineages within 48% of the studied individuals might be the outcome of an accelerated acquisition of beneficial mutations within hypermutators [11], [41]–[43]. Nonetheless, our examination of mutation dynamics of homopolymers provided a novel genome-wide perspective on the impact and potential advantage of differential mutagenesis associated with the hypermutator phenotype. Showing a clear exponential correlation between the rate of change and the size of the homopolymer, we confirmed homopolymers to be hotspots for differential mutagenesis, and we identified two homopolymer-containing genes to be preferentially mutated in hypermutators.

In conclusion, we have shown how collections of isolates of bacteria sampled from chronically infected patients constitute a valuable basis for studying evolution of pathogens *in vivo*, and our results facilitates comparative studies as sequencing datasets become increasingly available.

## Materials and Methods

### Bacterial strains and genome sequencing

The study encompasses 55 isolates of the *P. aeruginosa* DK2 clone type that were sampled over 38 years from 21 CF patients attending the Copenhagen Cystic Fibrosis Center at the University Hospital, Rigshospitalet (Figure 1). Isolation and identification of *P. aeruginosa* from sputum was done as previously described [44]. Sequencing of 45 of the isolates was previously reported by Yang *et. al.* [10] and Rau *et al.* [9]. Two of the previously sequenced isolates (CF333-1991 and CF510-2006) were re-sequenced together with ten new isolates on an Illumina HiSeq2000 platform generating 100-bp paired-end reads using a multiplexed protocol to an average coverage depth of 63–212 fold. Sequence reads from all isolates are deposited in the Short Read Archive under accession number ERP002277 (accession numbers for individual samples are provided in Table S4).

### Mutation detection and analysis

Reads were mapped against the *P. aeruginosa* DK2 reference genome (CF333-2007a; Genbank accession no. CP003149) using Novoalign (Novocraft Technologies) [45], and pileups of the read alignments were produced by SAMtools release 0.1.7 [46]. Single nucleotide polymorphisms were called by the varFilter algorithm in SAMtools in which minimum SNP coverage was set to 3 (samtools.pl varFilter -d 3 -D 10000). Only SNP calls with quality scores (Phred-scaled probability of sample reads being homozygous reference) of at least 50 (*i.e. P*≤10^−5^) were retained. Microindels were extracted from the read pileup by the following criteria; (1) quality scores of at least 500, (2) root-mean-square (RMS) mapping qualities of at least 25, and (3) support from at least one fifth of the covering reads. The false-negative rates were found to be 2% and 3% by *in silico* introduction of random base-substitutions and microindels (lengths 1–10 bp), respectively. To avoid false-positives, the reference genome was re-sequenced by Illumina sequencing to exclude polymorphisms caused by errors in reference assembly. Also, Illumina re-sequencing of CF333-1991 confirmed all the SNPs (and found no other SNPs) that were previously reported for this isolate by use of pyrosequencing [10]. Indeed, the confirmation by re-sequencing of CF333-1991 and the fact that many isolates are only discriminated by a few mutations verify that our genomic analysis has a very low false-positive rate.

A maximum-parsimonious phylogenetic analysis was used to predict the relationship and mutational events among the clones of the DK2 clone type. The tree consistency index (CI = *m*/*s*) was calculated as the minimum number of changes (*m*) divided by the number of changes required on the tree (*s*). The CI will equal 1 when there is no homoplasy.

For the calculation of average distances of the 15 CF333 isolates to their line of descent, the line of descent was defined as the direct lineage from the most recent common ancestor (MRCA) of all 15 isolates until the MRCA of the three most recently sampled isolates (CF333-2007a, CF333-2007b, CF333-2007c).

To provide the most accurate estimates of the relative homopolymer mutation rates in the *mutS/mutL* MMR-deficient sub-lineages, we calculated the rates per *mutS/mutL* MMR-deficiency caused SNP. This corrected count of SNPs were found by subtracting the fraction of SNPs expected to have accumulated due to the normal underlying mutation rate, *i.e.* SNPs not caused by the *mutS/mutL* MMR-deficiency. For this purpose a 2∶1 transition to transversion ratio was assumed for the normal background mutation rate. This means that the SNP count of hypermutator branch “KD” composed of 2,534 SNPs (Table 1), hereof 29 transversions, was corrected to 2,447 *mutS/mutL* MMR-deficiency caused SNPs. All results and conclusions were unaffected from this correction.

### Bayesian evolutionary analysis

Bayesian analysis of evolutionary rates and divergence times was performed using BEAST v1.7.2 [47]. BEAST was run with isolate CF510-2006 as an outgroup [9] and the following user-determined settings; a lognormal relaxed molecular clock model which allows rates of evolution to vary amongst the branches of the tree, and a general time-reversible substitution model with gamma correction. Results were produced from three independent chains of 50 million steps each, sampled every 5,000 steps. The first 5 million steps of each chain were discarded as a burn-in. The results were combined, and the maximum clade credibility tree was generated (using LogCombiner and TreeAnnotator programs from the BEAST package, respectively). The effective sample-sizes (ESS) of all parameters were >500 as calculated by Tracer v1.5 (available from http://beast.bio.ed.ac.uk/Tracer), which was also used to calculate 95% HPD confidence intervals of the mutation rate (*i.e.* an interval in which the modeled parameter resides with 95% probability). The root of the tree was predicted to be in year 1943 (95% HPD, 1910–1962). Note, as this estimate is based on isolates primarily sampled after year 1980, the same accumulation rate of SNPs might not hold true for the evolution of the DK2 clone type before 1980.

## Supporting Information

Figure S1 Distribution of SNPs accumulated in the branch leading to CF224-2002a. Genomic overview of the distribution of SNPs accumulated in the branch leading to isolate CF224-2002a according to the predicted phylogeny in Figure 2. The positions of the two genomic regions, in which the majority of the SNPs were found, and the remaining 8 SNPs are all indicated by text. The results provide evidence of two events in which imported DNA have recombined into the chromosome of CF224-2002a. The high density of polymorphisms suggests the imported DNA to origin from a *P. aeruginosa* strain(s) unrelated to the DK2 clone type. No genes related to mobilization and transfer of DNA was present within a 10 kb range of the predicted boundaries of the horizontally transferred regions, so we have no evidence for elements specialized for horizontal gene transfer to play a role.
(EPS)

Figure S2 Mutation rates of homopolymers in intragenic and intergenic regions. Rates of mutation of homopolymers of different sizes are shown for intragenic and intergenic homopolymers, respectively. The rates are averages of the seven DK2 sub-lineages evolving with a *mutS/mutL* MMR-deficiency. The rates were calculated as the number of observed indels per homopolymer per *mutS/mutL* MMR-deficiency caused SNP (see Materials and Methods).
(EPS)

Figure S3 Bayesian phylogenetic reconstruction and divergence date estimates of the *P. aeruginosa* DK2 clones. Bayesian statistics were used to estimate the divergence times of predicted ancestors. The tree is based on 7,326 unique SNPs identified from whole-genome sequencing. Circles labeled A1, A2, B1, B2, C1, and C2, respectively, denotes the position of the first and last genotype of each of the DK2 sub-lineages A, B, and C which were observed to have infected patient CF173.
(EPS)

Figure S4 Maximum-parsimonious reconstruction of the phylogeny of the *P. aeruginoisa* DK2 clones. The phylogenetic tree is based on 8,530 mutations (SNPs and indels) identified from whole-genome sequencing. Outlier isolate CF510-2006 (not shown) was used as an outgroup to root the tree. Italic letters indicate branch names, and lengths of branches are proportional to the number of mutations. The specific mutations that have accumulated during each specific branch are listed in Table S5 (SNPs) and Table S6 (indels).
(EPS)

Table S1 Prevalence of homopolymers in essential genes and genes functionally related to the composition of the cell envelope.
(DOCX)

Table S2 Full list of pathoadaptive genes (n = 65).
(XLSX)

Table S3 Non-synonymous mutations in genes *rpoB*, *gyrA* and *gyrB*. For each of the mutations, we have listed studies of our knowledge to have shown or indicated the specific mutation to confer resistance against an antibiotic.
(DOCX)

Table S4 List of sequenced samples and corresponding Sequence Read Archive (SRA) accession numbers.
(XLSX)

Table S5 Full list of SNPs.
(XLSX)

Table S6 Full list of indels.
(XLSX)

Text S1 Mutational signature of genetic drift.
(DOCX)
