# Supplementary material for: Genome Analysis of a Transmissible Lineage of Pseudomonas aeruginosa Reveals Pathoadaptive Mutations and Distinct Evolutionary Paths of Hypermutators
Source: PLoS Genet. 2013 Sep 5;9(9):e1003741. doi: 10.1371/journal.pgen.1003741 (PMC3764201; doi:10.1371/journal.pgen.1003741)
Supplement: Table S1 — Prevalence of homopolymers in essential genes and genes functionally related to the composition of the cell envelope. (DOCX) [file pgen.1003741.s005.docx]

**Table S1: Prevalence of homopolymers in essential genes and genes functionally related to the composition of the cell envelope.**

| Genes | Total number of genes | Genes containing large homopolymers (≥7xN) | Proportion | Fisher's Exact Test one-tailed (*P*-value) |
| --- | --- | --- | --- | --- |
| All | 5677 | 85 | 0.015 |  |
| Essential | 335 | 1 | 0.003 | 0.0370 |
| Cell wall / LPS / Capsule^a^ | 179 | 9 | 0.050 | 0.0017 |

^a^ Genes belonging to PseudoCap Function Class “Cell wall / LPS / Capsule”
