# Supplementary material for: Genome Analysis of a Transmissible Lineage of Pseudomonas aeruginosa Reveals Pathoadaptive Mutations and Distinct Evolutionary Paths of Hypermutators
Source: PLoS Genet. 2013 Sep 5;9(9):e1003741. doi: 10.1371/journal.pgen.1003741 (PMC3764201; doi:10.1371/journal.pgen.1003741)
Supplement: Table S3 — Non-synonymous mutations in genes rpoB, gyrA and gyrB. For each of the mutations, we have listed studies of our knowledge to have shown or indicated the specific mutation to confer resistance against an antibiotic. (DOCX) [file pgen.1003741.s007.docx]

**Table S3: Non-synonymous mutations in genes *rpoB*, *gyrA* and *gyrB*.**

| Gene | Nucleotide change | Amino acid change | Studies describing the mutation |
| --- | --- | --- | --- |
| *rpoB* | G713A | G238D |  |
|  | A1364G | N455S |  |
|  | A1487G | D496G |  |
|  | A1562G | D521G | [1,2,3,4] |
|  | C1591A | H531N | [3] |
|  | A1592T | H531L | [2,3,4] |
|  | G3376A | V1126I |  |
|  | A3718G | T1240A |  |
| *gyrA* | C248T | T83I | [5] |
|  | G259T | D87Y | [6] |
|  | G259A | D87N | [7,8,9] |
|  | A260G | D87S | [10] |
|  | G1843C | A615P |  |
| *gyrB* | G943A | D315N |  |
|  | C1397T | S466F | [11] |
|  | G1404T | E468D | [12] |

For each of the mutations, we have listed studies of our knowledge to have shown or indicated the specific mutation to confer resistance against an antibiotic.

1. Escobar-Paramo P, Gougat-Barbera C, Hochberg ME (2012) Evolutionary dynamics of separate and combined exposure of Pseudomonas fluorescens SBW25 to antibiotics and bacteriophage. Evol Appl 5: 583-592.

2. Hall AR, Iles JC, MacLean RC (2011) The fitness cost of rifampicin resistance in Pseudomonas aeruginosa depends on demand for RNA polymerase. Genetics 187: 817-822.

3. Weigand MR, Sundin GW (2012) General and inducible hypermutation facilitate parallel adaptation in Pseudomonas aeruginosa despite divergent mutation spectra. Proc Natl Acad Sci U S A 109: 13680-13685.

4. MacLean RC, Buckling A (2009) The distribution of fitness effects of beneficial mutations in Pseudomonas aeruginosa. PLoS Genet 5: e1000406.

5. Cabot G, Ocampo-Sosa AA, Dominguez MA, Gago JF, Juan C, et al. (2012) Genetic markers of widespread extensively drug-resistant Pseudomonas aeruginosa high-risk clones. Antimicrob Agents Chemother 56: 6349-6357.

6. Komp Lindgren P, Karlsson A, Hughes D (2003) Mutation rate and evolution of fluoroquinolone resistance in Escherichia coli isolates from patients with urinary tract infections. Antimicrob Agents Chemother 47: 3222-3232.

7. Gullberg E, Cao S, Berg OG, Ilback C, Sandegren L, et al. (2011) Selection of resistant bacteria at very low antibiotic concentrations. PLoS Pathog 7: e1002158.

8. Wang H, Dzink-Fox JL, Chen M, Levy SB (2001) Genetic characterization of highly fluoroquinolone-resistant clinical Escherichia coli strains from China: role of acrR mutations. Antimicrob Agents Chemother 45: 1515-1521.

9. Cui S, Li J, Sun Z, Hu C, Jin S, et al. (2009) Characterization of Salmonella enterica isolates from infants and toddlers in Wuhan, China. J Antimicrob Chemother 63: 87-94.

10. Streicher EM, Bergval I, Dheda K, Bottger EC, Gey van Pittius NC, et al. (2012) Mycobacterium tuberculosis population structure determines the outcome of genetics-based second-line drug resistance testing. Antimicrob Agents Chemother 56: 2420-2427.

11. Llanes C, Kohler T, Patry I, Dehecq B, van Delden C, et al. (2011) Role of the MexEF-OprN efflux system in low-level resistance of Pseudomonas aeruginosa to ciprofloxacin. Antimicrob Agents Chemother 55: 5676-5684.

12. Pasca MR, Dalla Valle C, De Jesus Lopes Ribeiro AL, Buroni S, Papaleo MC, et al. (2012) Evaluation of fluoroquinolone resistance mechanisms in Pseudomonas aeruginosa multidrug resistance clinical isolates. Microb Drug Resist 18: 23-32.
