# Supplementary material for: Genome Analysis of a Transmissible Lineage of Pseudomonas aeruginosa Reveals Pathoadaptive Mutations and Distinct Evolutionary Paths of Hypermutators
Source: PLoS Genet. 2013 Sep 5;9(9):e1003741. doi: 10.1371/journal.pgen.1003741 (PMC3764201; doi:10.1371/journal.pgen.1003741)
Supplement: Text S1 — Mutational signature of genetic drift. (DOCX) [file pgen.1003741.s011.docx]

**Text S1: Mutational signature of genetic drift**

The SNPs were distributed equally in the intergenic (12%) and intragenic (88%) regions, respectively (89.32 % of genome is coding). The dN/dS ratio can be used a measure of the selection pressure acting on the protein coding genome [1]. A general observed signature of genetic drift (dN/dS=0.66) was significantly more predominant in sub-lineages that had evolved as hyper-mutators (dN/dS=0.69) relative to sub-lineages with no indications of hyper-mutation or HGT (dN/dS=0.92; Fisher’s exact test, *P*=0.002). Furthermore, the SNPs that had accumulated in CF224-2002a as a consequence of horizontal gene transfer, displays a even more stringent selection coefficient of dN/dS = 0.07 which is close to what have previously been reported for homologue DNA between unrelated strains of *P. aeruginosa* (dN/dS=0.1) [2].

There was a relative underrepresentation of intragenic microindels (65%) which can be explained by a general negative selection for the deleterious effects of microindels causing frame-shifts, hence, the observation is in agreement with the overall signature of genetic drift. Accordingly, we as expected observe relative more indels whose lengths are a multiple of three (*i.e.* maintaining the reading frame) in intragenic regions versus intergenic regions (16% and 7%, respectively; Fisher’s exact test, *P*=1.2×10^-6^).

1. Yang Z, Bielawski JP (2000) Statistical methods for detecting molecular adaptation. Trends Ecol Evol 15: 496-503.

2. Smith EE, Buckley DG, Wu Z, Saenphimmachak C, Hoffman LR, et al. (2006) Genetic adaptation by Pseudomonas aeruginosa to the airways of cystic fibrosis patients. Proc Natl Acad Sci U S A 103: 8487-8492.
